# Trajectory-based identification of cognitive-performance phenotypes across adulthood from psychophysiological testing

**DOI:** 10.3389/fnagi.2026.1898458

**Published:** 2026-08-11

**Authors:** Alexander E. Zapryalov, Sergey V. Stasenko, Nadezhda A. Chumankina, Danila D. Shashnin, Maria V. Vedunova

**Affiliations:** 1 Institute of Biology and Biomedicine, Lobachevsky State University of Nizhniy Novgorod, Nizhny Novgorod, Russia; 2 Moscow Center for Advanced Studies, Moscow, Russia

**Keywords:** adult individual differences, cognitive performance, elastic principal graph, psychophysiological testing, trajectory inference, unsupervised structure learning

## Abstract

**Background:**

Multidimensional psychophysiological batteries reveal substantial inter-individual variation in processing speed, accuracy, memory, executive control, and visuospatial performance. Because the present sample is predominantly young to middle-aged, the analysis is framed as adult cognitive-performance phenotyping rather than identification of clinical cognitive-aging stages.

**Methods:**

We analyzed a cross-sectional convenience sample of 1,117 adults (age 18–78 years; mean 31.4 ± 12.0 years; 65.8% women) assessed with a web-based psychophysiological battery. Forty-two cognitive, psychomotor, demographic, anthropometric, lifestyle, and self-reported health variables were standardized after singular-value-decomposition imputation of one missing BMI value. DANCo and local PCA estimated intrinsic dimensionalities of 5.83 and 3.82, respectively. Six principal components (39.24% cumulative variance; seventh-component increment 3.67%) were used to fit an elastic principal tree. Between-branch differences were evaluated by Kruskal–Wallis tests with Holm-adjusted Dunn comparisons or chi-square tests, with effect sizes. Assignment reproducibility was evaluated in 100 random 80% subsamples projected onto the fixed reference tree. The workflow is an unsupervised statistical/geometric structure-learning analysis rather than supervised prediction; no train/test predictive model or learned longitudinal dynamics are claimed.

**Results:**

Three connected branch profiles were identified: Cluster 0 (*n* = 781, 69.9%), a high-accuracy reference profile; Cluster 1 (*n* = 98, 8.8%), a rapid-processing profile with domain-specific visuospatial variability; and Cluster 2 (*n* = 238, 21.3%), a slower, lower-accuracy profile enriched for older age and positive insomnia/disease indicators. Moderate-to-large effects were observed for Stroop-4 speed (η^2^ = 0.195), Stroop-4 accuracy (η^2^ = 0.194), figure-shape accuracy (η^2^ = 0.240), figure-shape-color accuracy (η^2^ = 0.251), and figure-shape-position accuracy (η^2^ = 0.253; all *p* < 0.0001). Fixed-tree projections reproduced assignments with mean adjusted Rand index, normalized mutual information, and raw agreement of 1.000 ± 0.000. In the additional sensitivity analyses, exclusion of the binary variables changed the solution from three to nine clusters (ARI = 0.1203), and reduction from six to four PCs changed it from three to seven clusters (ARI = 0.1736). All binary features in the dataset are directly or indirectly related to cognitive functions or to the interpretation of psychophysiological test results. Removing the complete binary-variable block therefore changed not only the numerical feature space but also the substantive interpretation of the analysis, particularly because this block included age-associated lifestyle and health factors such as smoking, alcohol use, insomnia, and disease status.

**Conclusion:**

Elastic principal trees represent cognitive-performance profiles as connected branches and provide a graph-ordering coordinate unavailable from ordinary discrete clustering. The results are exploratory, cross-sectional, and non-diagnostic and should not be generalized to neurodegenerative aging without older, clinically characterized, longitudinal cohorts. Among the examined alternatives, the original full-feature six-PC solution was retained for interpretation because the alternative solutions produced additional very small clusters that could not be characterized reliably.

## 1 Introduction

Population aging has made preservation of cognitive health a major scientific and public-health priority. Dementia affects memory, thinking, behavior, and independence; the World Health Organization reported that 57 million people were living with dementia in 2021 and that nearly 10 million new cases occur annually (World Health Organization, 2025). Global estimates suggest that dementia prevalence may rise to approximately 152.8 million people by 2050, driven by demographic aging and population growth (Nichols et al., 2022). The 2024 Lancet Commission emphasized that a substantial proportion of dementia risk is potentially modifiable across the life course, including education, vascular factors, hearing and vision loss, depression, diabetes, physical inactivity, smoking, excessive alcohol use, and social isolation (Livingston et al., 2024). This creates a practical need for methods that can separate relatively healthy cognitive aging from potentially pathological or risk-enriched aging before severe impairment is clinically evident. Cognitive aging is not a uniform decline. Reviews of neurocognitive aging show that different cognitive systems follow different life-course curves, with processing speed and executive control often declining earlier than vocabulary or accumulated knowledge (Cohen et al., 2019; Grady, 2012; Harada et al., 2013). In normal aging, many individuals retain adequate everyday cognitive function despite measurable slowing in attention, mental flexibility, working memory, and visuospatial processing (Salthouse et al., 1995; Hartshorne et al., 2015). Large behavioral datasets show asynchronous peaks and declines across cognitive abilities (Hartshorne et al., 2015), and studies of visual working memory demonstrate broad age-related variability rather than a single universal trajectory (Brockmole et al., 2013). Working memory is especially relevant because it supports goal-directed behavior and integrates attention, memory, and executive control (Baddeley, 2012; Archer et al., 2018). The clinical boundary between healthy aging and abnormal cognitive decline is gradual. Mild cognitive impairment (MCI) is commonly conceptualized as measurable impairment in one or more cognitive domains that does not yet substantially impair daily independence but is associated with elevated dementia risk (Petersen, 2016). Traditional tools such as the Mini-Mental State Examination and Montreal Cognitive Assessment remain useful for screening (Nasreddine et al., 2005; Folstein et al., 1975), but they do not fully capture the multidimensional dynamics of cognitive aging or the heterogeneity of early decline. Cognitive reserve theory further suggests that education, lifestyle, and accumulated neural/behavioral resources can help some individuals maintain performance despite age-related brain changes or pathology (Stern, 2002). Executive control and reaction-time measures are particularly promising for distinguishing preserved from vulnerable aging. A 2025 systematic review of Stroop and Simon paradigms concluded that cognitive control is among the first functions to decline with age and that altered executive-control effects can distinguish healthy aging from cognitive impairment (Cipriani et al., 2025). A 2025 Stroop study comparing cognitively normal older adults and MCI patients found that MCI was associated with slower responses and lower accuracy under higher cognitive load, especially when interference inhibition was required (Zhu et al., 2025). These findings are directly relevant to datasets that include Stroop speeds, Stroop accuracies, arithmetic response times, visuospatial discrimination, and motor reaction time. Recent work also supports the use of digital and psychophysiological testing for cognitive-age estimation. Tyurina et al. (2025) used psychophysiological tasks measuring reaction time, cognitive conflict, short-term memory, verbal functions, color perception, and spatial perception to predict chronological age; their best regularized model achieved a mean absolute error of approximately 5.7 years and identified Stroop timing and task-related timing features as the strongest predictors (Tyurina et al., 2025). Digital cognitive assessments have also been evaluated for dementia staging and MCI detection: a 2025 retrospective study found that a digital cognitive battery predicted dementia stages with area-under-the-curve values ranging from 0.733 to 0.917 (Huynh et al., 2025), and a 2025 systematic review highlighted both the promise and methodological challenges of digital tools for MCI detection (Bonvino et al., 2025). Lifestyle and health factors can modulate cognitive trajectories. In a 2025 longitudinal analysis of 5084 middle-aged and older adults from CHARLS, cognitive trajectories were heterogeneous and current smoking was associated with greater odds of rapid decline compared with non-smoking (Li R. et al., 2025). Sleep disturbances are also increasingly recognized as modifiable cognitive-risk factors: an updated 2025 meta-analysis of longitudinal studies reported that insomnia, abnormal sleep duration, excessive daytime sleepiness, and poor sleep quality were associated with increased risk of cognitive decline or dementia (Zhang et al., 2025). Alcohol use remains controversial in observational studies, but a 2025 combined cohort and Mendelian-randomization analysis reported genetic evidence consistent with increasing dementia risk at higher alcohol exposure and no support for a protective effect of alcohol consumption (Topiwala et al., 2026). These recent findings justify incorporating self-reported health, sleep, and lifestyle variables when interpreting cognitive-aging profiles. The concept of cognitive age links cognitive performance to chronological aging while allowing individuals to deviate from the average age curve. Brain-age and cognitive-age studies suggest that the gap between predicted and chronological age can indicate accelerated or preserved aging (Tyurina et al., 2025; Miao et al., 2025; Statsenko et al., 2021; Li Z. et al., 2025; Lee et al., 2022). However, a single global prediction score can obscure heterogeneity: two individuals with similar predicted age may differ in whether slowing is driven by executive conflict, memory, visuospatial perception, motor reaction time, illness burden, insomnia, or lifestyle factors. Therefore, trajectory-based phenotyping may complement supervised age prediction by identifying groups and paths through a multidimensional cognitive-health landscape. Clinical trajectory analysis provides such a framework. Instead of assigning individuals to isolated clusters, elastic principal graph methods approximate a high-dimensional dataset by a branching tree, allowing patients or participants to be positioned along interpretable segments and ordered by pseudo-time (Golovenkin et al., 2020; Gorban et al., 2010, 2017). In clinical datasets, this approach has been used to reconstruct disease trajectories and identify phenotypes connected by intermediate states (Golovenkin et al., 2020; Bell et al., 2025).

The age composition of the present sample does not support a direct claim that the branches are stages of healthy versus pathological aging: 78.7% of participants belonged to two branches with mean ages below 30 years, and no formal cognitive diagnosis was available. We therefore use the ClinTrajan/ElPiGraph framework to characterize connected adult cognitive-performance profiles. Graph distance is treated as an inferred phenotypic coordinate, not calendar time or demonstrated biological progression. The specific objectives were to (i) identify connected profiles in the multidimensional psychophysiological dataset, (ii) quantify the variables distinguishing the branches, (iii) test between-branch differences with multiplicity control and effect sizes, and (iv) evaluate the reproducibility of branch assignment under repeated 80% subsampling and projection onto a fixed reference tree. Our descriptive hypothesis was that the branch-based profiles would differ in processing speed and accuracy, particularly in executive-control and visuospatial tasks. PCA and elastic principal-graph fitting are treated here as unsupervised statistical/geometric structure-learning procedures, not as supervised prediction. Accordingly, robustness is assessed through node-count analysis, projection onto a fixed reference tree, removal of the binary-variable block, and comparison of four- and six-PC solutions rather than through learning curves or train/test predictive performance.

## 2 Materials and methods

### 2.1 Study design and dataset

This cross-sectional study analyzed psychophysiological, demographic, anthropometric, lifestyle, and health-related variables to identify adult cognitive-performance profiles and their associations with age. The analytical dataset included 1117 observations and 42 variables. The participant identifier was used only for technical indexing and was excluded from the analytical feature space.

The study focused on detecting data-driven cognitive-performance profiles, with particular attention to branch differences in speed, accuracy, age, disease burden, insomnia, and lifestyle indicators. The analytical strategy was based on trajectory inference with elastic principal graphs, following the ClinTrajan/ElPiGraph concept of representing cross-sectional clinical data as a pseudo-diachronic landscape of branching trajectories (Golovenkin et al., 2020; Bell et al., 2025).

The analytical export comprised de-identified records collected with a web-based psychophysiological testing platform developed within the Lobachevsky University research program. The available export did not retain ethnicity, exact recruitment dates, medication data, formal neurological or psychiatric diagnoses, or a prospective exclusion log; the cohort should therefore be regarded as a convenience adult sample rather than a population-representative aging cohort. All records in the final analytical export with a valid anonymous identifier and test results were included. Participants ranged from 18 to 78 years (mean 31.4 ± 12.0); 735 (65.8%) were women. One BMI value was missing (0.09%), while all other analytical fields were complete (Table 1). The study was approved by the Ethics Committee of Lobachevsky University (Protocol No. 7, 25 October 2025), and all participants provided informed consent.

**Table 1:** Full-sample characteristics before branch assignment.

Characteristic	Full sample (*N* = 1, 117)
Age, years	31.4 ± 12.0 (range 18–78)
Women, *n* (%)	735 (65.8)
Left-handed, *n* (%)	72 (6.4)
Secondary/other rather than higher education, *n* (%)	119 (10.7)
Degree indicator present, *n* (%)	108 (9.7)
BMI, kg/m^2^	24.0 ± 4.8 (*n* = 1, 116)
Current smoking indicator, *n* (%)	156 (14.0)
Alcohol-use indicator, *n* (%)	224 (20.1)
Insomnia indicator, *n* (%)	202 (18.1)
Disease indicator, *n* (%)	255 (22.8)

### 2.2 Variables and cognitive testing measures

The dataset included variables reflecting several cognitive and psychomotor domains. Executive function and cognitive interference were assessed using Stroop-task indicators. Arithmetic processing was represented by measures of task performance and response characteristics in mathematical tasks. Memory-related variables reflected short-term and working-memory performance. Visual–spatial and perceptual functions were assessed using figure-position, figure-shape, and related visual tasks. Additional measures included Raven-type reasoning, Munsterberg word-search performance, maze-task indicators, word-sequence performance, logic-task performance, motor reaction measures for the left and right hands, interhemispheric differences, and balance-related indicators.

Demographic and health-related variables included age, sex, handedness, education, degree status, height, weight, body mass index (BMI), smoking, alcohol use, insomnia, and disease status. Variable-type detection classified 33 variables as continuous and 9 as binary. Binary variables included Logic, Sex, Handedness, Education, Degree, Smoking, Alcohol, Insomnia, and Diseases.

Sex was coded 0 = male and 1 = female; handedness 0 = right and 1 = left; education 0=higher education (bachelor, specialist, or master's level) and 1 = secondary, vocational, basic, or other education; smoking 0 = no and 1 = yes; alcohol 0 = no and 1 = any reported use; insomnia 0 = no and 1 = yes; and diseases 0 = no self-reported disease indicator and 1 = indicator present. Degree and Logic were retained as source 0/1 indicators, where 1 denotes the presence/success indicator recorded by the source instrument. These variables are questionnaire or task indicators rather than verified diagnoses. In particular, the Diseases variable does not identify disease type, severity, medication, or duration, and Insomnia is not a validated clinical insomnia scale. A complete coding dictionary, including interpretation limitations, is provided in Supplementary Table S3.

### 2.3 Data preprocessing

Before trajectory analysis, the dataset was inspected for correlations among variables. Spearman correlation analysis was used to evaluate monotonic associations between features and to identify potentially redundant variables. Correlation matrices were generated for the complete feature set, and strongly correlated pairs were defined using a threshold of 0.5.

The dataset contained mixed variable types, including continuous and binary indicators. Therefore, preprocessing followed a quantification strategy suitable for clinical and psychophysiological datasets with heterogeneous variable scales. Variables were transformed into a numerical representation appropriate for multivariate modeling. Missing values were imputed using an SVD-based imputation procedure. After imputation, the data were transformed back to the original variable scales for descriptive interpretation.

For trajectory construction, the quantified feature matrix was standardized so that variables with larger numerical ranges would not dominate the analysis. Original-scale variables were retained for interpretation of cluster means, distributions, and cognitive-performance profiles.

Only one value (BMI) was imputed. SVD imputation was retained because missingness was minimal and the procedure was already integrated into the ClinTrajan preprocessing workflow; no substantive conclusion depends on this single imputed value. Standard PCA is not ideal for mixed continuous and binary data. It was retained for compatibility with the elastic-graph pipeline and because all features were variance-standardized. Binary imbalance may reduce the contribution of rare indicators and obscure minority patterns; binary variables were therefore also tested separately with chi-square procedures.

### 2.4 Dimensionality reduction

The intrinsic dimensionality of the dataset was estimated before principal-tree construction. Two complementary methods, DANCo (Ceruti et al., 2014) and local PCA, indicated that the data had a low-dimensional structure. Based on these estimates, the standardized feature matrix was reduced to six principal components using principal component analysis with a full singular value decomposition solver. This six-dimensional representation was used as the input space for nonlinear trajectory modeling.

DANCo estimated intrinsic dimensionality as 5.83, whereas local PCA gave a mean estimate of 3.82. Six components were selected as a conservative integer representation covering the DANCo estimate. Together they explained 39.24% of standardized variance; the seventh component added 3.67 percentage points. The three largest absolute loadings were: PC1, age (0.277), memory speed (0.248), and Munsterberg word count (−0.246); PC2, weight (0.510), height (0.378), and sex (−0.378); PC3, figure-shape-color accuracy (0.446), figure-shape-position accuracy (0.441), and figure-shape accuracy (0.424); PC4, left and right motor-reaction accuracy (0.299 and 0.295) and Stroop-4 speed (−0.261); PC5, memory accuracy (0.334), Stroop-4 speed (0.323), and left motor-reaction speed (0.322); and PC6, left and right motor-reaction speed (0.381 and 0.356) and sex (−0.302). The complete loading matrix is provided as Supplementary Table S1.

### 2.5 Sensitivity analyses for binary variables and PCA dimensionality

To examine whether the binary variables influenced the PCA representation and the principal-tree structure, all binary variables were removed and the analysis was repeated. This was treated as a sensitivity perturbation rather than an alternative primary specification. All binary features in the dataset are directly or indirectly related to cognitive functions or to the interpretation of psychophysiological test results. Removing the complete binary-variable block therefore changes not only the numerical feature space but also the substantive interpretation of the analysis, particularly because this block includes age-associated lifestyle and health factors such as smoking, alcohol use, insomnia, and disease status. The principal tree obtained from the full dataset was compared with the tree obtained without binary variables. A contingency matrix was constructed to show the distribution of participants between the original and new clusters in absolute numbers and as column percentages. Agreement between the two partitions was quantified with the adjusted Rand index (ARI).

Sensitivity to the number of retained principal components was evaluated by comparing the reference six-PC analysis with an analysis based on four PCs. Six PCs explained 39.2% of the variance, whereas four PCs explained 31.3%. The seventh component added 3.7% of explained variance and subsequent components added still less; therefore, the sensitivity analysis evaluated a reduction to four PCs rather than an increase beyond six. The six-PC and four-PC cluster assignments were compared using contingency matrices and ARI.

### 2.6 Elastic principal tree construction

The nonlinear geometry of the dataset was approximated using elastic principal graph analysis. An elastic principal tree with 40 nodes was fitted to the six-dimensional PCA representation of the data. The model was constructed using penalized elastic energy minimization with the following parameters: alpha = 0.01, Mu = 0.1, Lambda = 0.05, and FinalEnergy = Penalized. After construction, the tree was pruned and terminal leaves were extended to better represent peripheral observations.

In the ElPiGraph objective, data approximation is balanced against elastic regularization. Lambda is the edge-stretching elasticity coefficient; it regularizes total graph length and promotes an approximately equidistant distribution of nodes. Mu is the star-bending elasticity coefficient; it penalizes deviation of a branching star from a harmonic configuration, defined by coincidence of the central node with the mean position of its leaves. alpha enters the degree-dependent penalized edge elasticity and increases the penalty for edges incident to higher-order branching nodes, thereby discouraging unsupported topological complexity (Golovenkin et al., 2020; Albergante et al., 2020). The selected combination was used by Golovenkin et al. in their clinical trajectory analyses and is reproduced in the official ClinTrajan tutorial as close to the default regime. It is therefore treated here as a literature-grounded reference configuration, not a universal optimum and not a *post hoc* choice made to obtain three branches.

**Figure 1:**
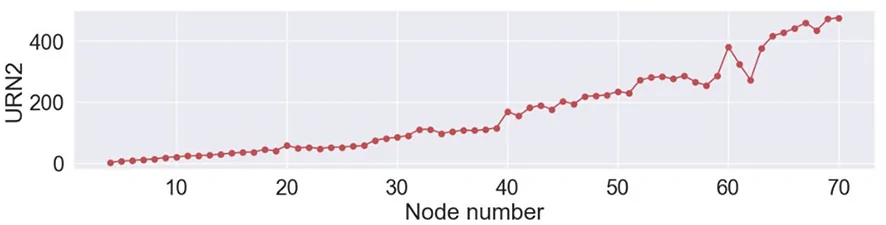
Node-count sensitivity. The unregularized node-complexity criterion (URN2) increases gradually at lower node counts and begins a steeper rise around 40 nodes, supporting selection of the 40-node reference resolution before marked complexity inflation.

Trees with increasing node counts were compared using the unregularized node-complexity criterion (URN2). Complexity increased gradually at lower resolutions and showed a substantially steeper rise at approximately 40 nodes (Figure 1); 40 nodes was selected as the elbow immediately before marked complexity inflation. Pruning and leaf extension produced 43 nodes in the final reference graph without changing the three principal non-branching segments.

The resulting tree was interpreted as a multidimensional adult cognitive-performance landscape. In this framework, each participant occupies a position on the tree according to similarity across cognitive, demographic, lifestyle, and health-related variables. Tree branches represent non-linear gradients of cognitive-performance profiles rather than predefined diagnostic categories.

### 2.7 Cluster assignment

Participants were assigned to clusters according to the non-branching segments of the elastic principal tree. This branch-based partitioning produced three main clusters. Each participant was also assigned to the nearest tree node using Euclidean proximity in the reduced feature space.

Cluster-level descriptive statistics were calculated for all variables, including mean, standard deviation, variance, minimum, quartiles, median, upper percentiles, maximum, skewness, kurtosis, and missing-value frequency. Cluster interpretation was performed using original-scale variables. Continuous variables were interpreted as mean cognitive or physiological values, whereas binary variables were interpreted as coded proportions.

### 2.8 Root-node selection, trajectory definition, and graph-distance estimation

**Figure 2:**
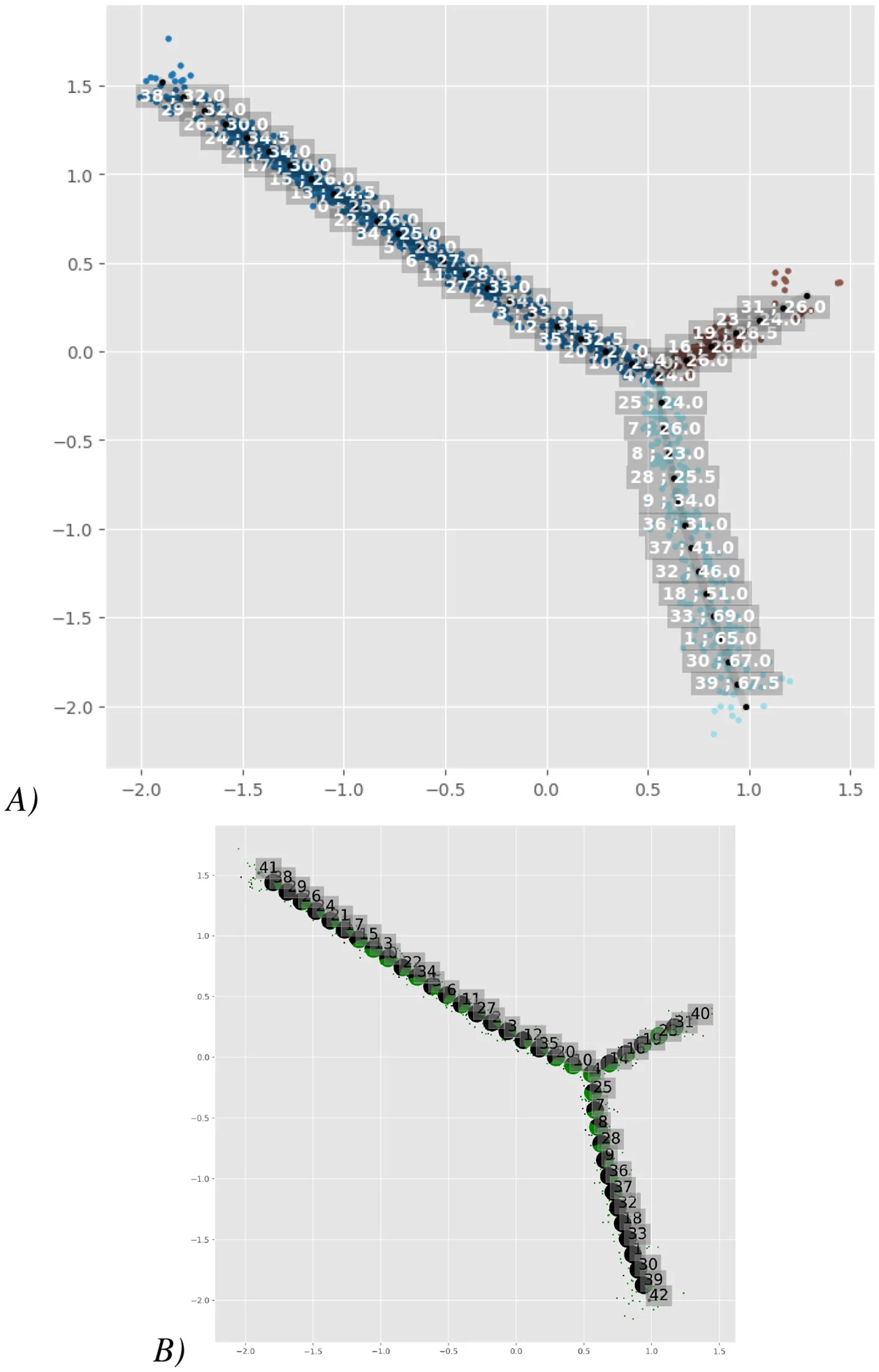
Root-node selection from complementary age distributions. **(A)** Median age at each tree node; labels report the node number and median age. Comparison of the three branches showed that the right branch had the lowest median age. **(B)** Proportion of participants younger than 25 years at each node; green sectors indicate the relative proportion of younger participants. The concentration of younger participants along the same right branch and its terminal region corroborated the median-age result. These age-based findings were considered together with the absence of coded smoking, alcohol-use, insomnia, and disease indicators in the candidate root region on the right branch; these age-associated lifestyle and health factors are related to cognitive functions and psychophysiological test performance. Node 40, the terminal node at the outer end of this youngest and most favorable branch, was selected as the root and as the starting point for constructing paths toward the other terminal branches. Root selection was performed after graph fitting and only set the orientation and origin of graph distance; it did not alter the tree or branch assignments.

The tree topology was first examined to identify branching points and terminal branches. Root selection combined age and the binary lifestyle/health profile of the candidate starting region. Median age was compared across the three branches (Figure 2A), and the node-wise proportions of participants younger than 25 years were examined (Figure 2B). The right branch had the lowest median age, while Panel B showed a concentration of younger participants along the same branch and its terminal region. The candidate root region on the right branch was also characterized by the absence of coded smoking, alcohol-use, insomnia, and disease indicators. Because these age-associated lifestyle and health factors are related to cognitive functions and psychophysiological test performance, their absence, together with the youngest age profile, supported identification of the right branch as the starting branch. Node 40 is the terminal node at the outer end of this branch and was selected as the root because it provides a natural starting point from which paths toward the terminal nodes of the other branches can be constructed.

After root-node selection, trajectories were defined as graph paths from node 40 to the terminal nodes. Shared internal segments represent portions of the tree common to participants before the branches separate, whereas terminal segments represent branch-specific cognitive-performance profiles. Graph distance was calculated as geodesic distance from node 40 along each path and was used as a phenotypic ordering coordinate. Because the root was selected using age, this coordinate should not be interpreted as an independent age association, chronological time, or demonstrated biological progression. Root selection was performed after the principal tree had been fitted and did not alter its topology, node positions, or branch assignments; it only fixed the orientation and origin of graph distance.

One trajectory corresponding to a single non-informative branch was excluded from further descriptive analysis. The two remaining root-to-terminal trajectories were retained for analysis of variable changes along graph distance.

### 2.9 Association of variables with pseudo-time

To determine which variables varied along each graph path, cognitive, demographic, lifestyle, and health-related features were modeled as functions of pseudo-time. Continuous variables were analyzed using nonlinear regression along the trajectory, whereas binary variables were modeled using an appropriate binary-response regression approach.

**Figure 3:**
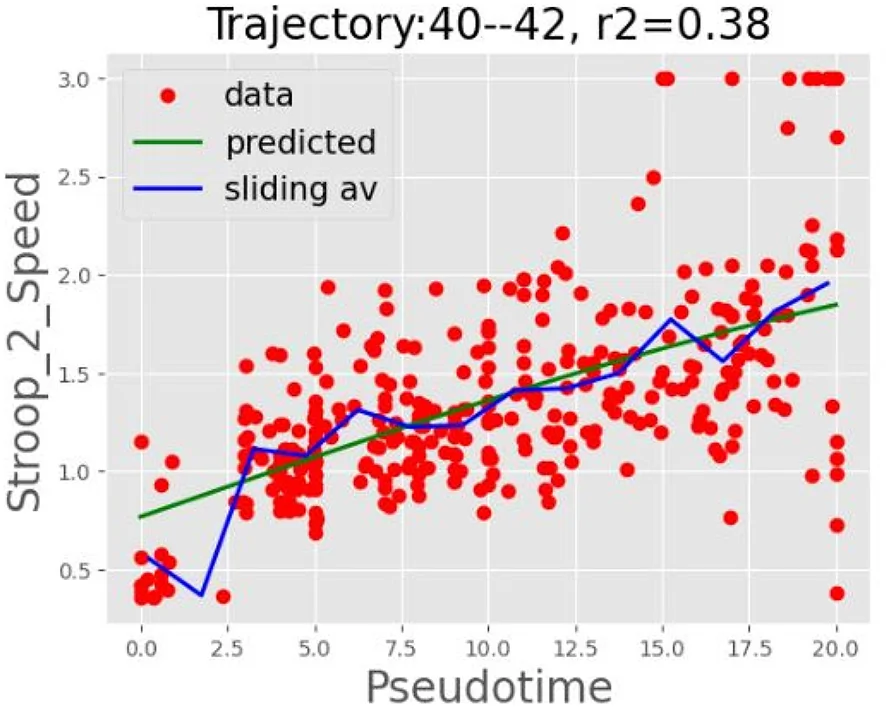
Example of pseudo-time regression for Stroop_2_Speed along trajectory 40–42. Red points represent participant data, the green line is the predicted regression curve, and the blue line is the sliding average. The coefficient of determination indicates the quality of approximation. The scatter also shows local sample density. The curves are descriptive summaries of cross-sectional graph ordering and are not longitudinal estimates or inferential uncertainty bands.

Regression curves along graph distance were treated as exploratory descriptive visualizations rather than inferential tests. The previous unequal retention thresholds (*R*^2^>0.5 for most variables and *R*^2^>0.1 for selected lifestyle variables) were removed as criteria for declaring relevance. Fit statistics are reported in the analysis output without selective significance claims. Figure 3 is retained only to illustrate the smoothing procedure; its *R*^2^ = 0.38 is explicitly below the former 0.5 threshold. Because the pseudo-time smooths were not used as hypothesis tests, no multiple-comparison correction was applied to them; Holm adjustment was applied to the formal between-branch comparisons described below.

### 2.10 Statistical and computational analysis

All analyses were conducted in Python using standard scientific-computing and data-analysis libraries, including pandas, NumPy, SciPy, scikit-learn, Matplotlib, Seaborn, ElPiGraph, ClinTrajan, scikit-dimension, and lifelines (Zhou, 2015). No supervised predictive model was trained; therefore, train/test prediction metrics and learning curves are not applicable to this analysis. The final outputs included correlation matrices, principal-tree coordinates, node assignments, branch labels, pseudo-time trajectories, and cluster-level descriptive statistics.

Continuous variables were compared across branches with Kruskal–Wallis tests because many distributions were skewed or bounded. Significant omnibus tests were followed by Dunn pairwise comparisons with Holm adjustment. Effect size was summarized by eta-squared derived from the Kruskal–Wallis statistic. Binary variables were evaluated with chi-square tests and Cramer's *V*, with Holm-adjusted pairwise comparisons where appropriate. Complete results are reported in Supplementary Table S2.

A naive bootstrap that refits a new elastic tree in each sample is not directly label-identifiable for this branch-based procedure because branches may change node numbering, split, merge, or rotate. We therefore evaluated the reproducibility of the operational assignment rule. One hundred random subsamples, each containing 80% of observations (*n* = 893), were drawn without replacement and projected onto the final six-dimensional reference tree. Each observation was assigned to its nearest graph node and non-branching segment and compared with its original label using adjusted Rand index (ARI), normalized mutual information (NMI), and raw agreement. This assesses assignment stability conditional on the fixed tree; it does not prove that independently refitted graph topologies are identical. Analysis code, source data, and notebooks are available at https://github.com/AlexanderZapryalov/cognitive-tests.

## 3 Results

### 3.1 Feature structure and distributions

**Figure 4:**
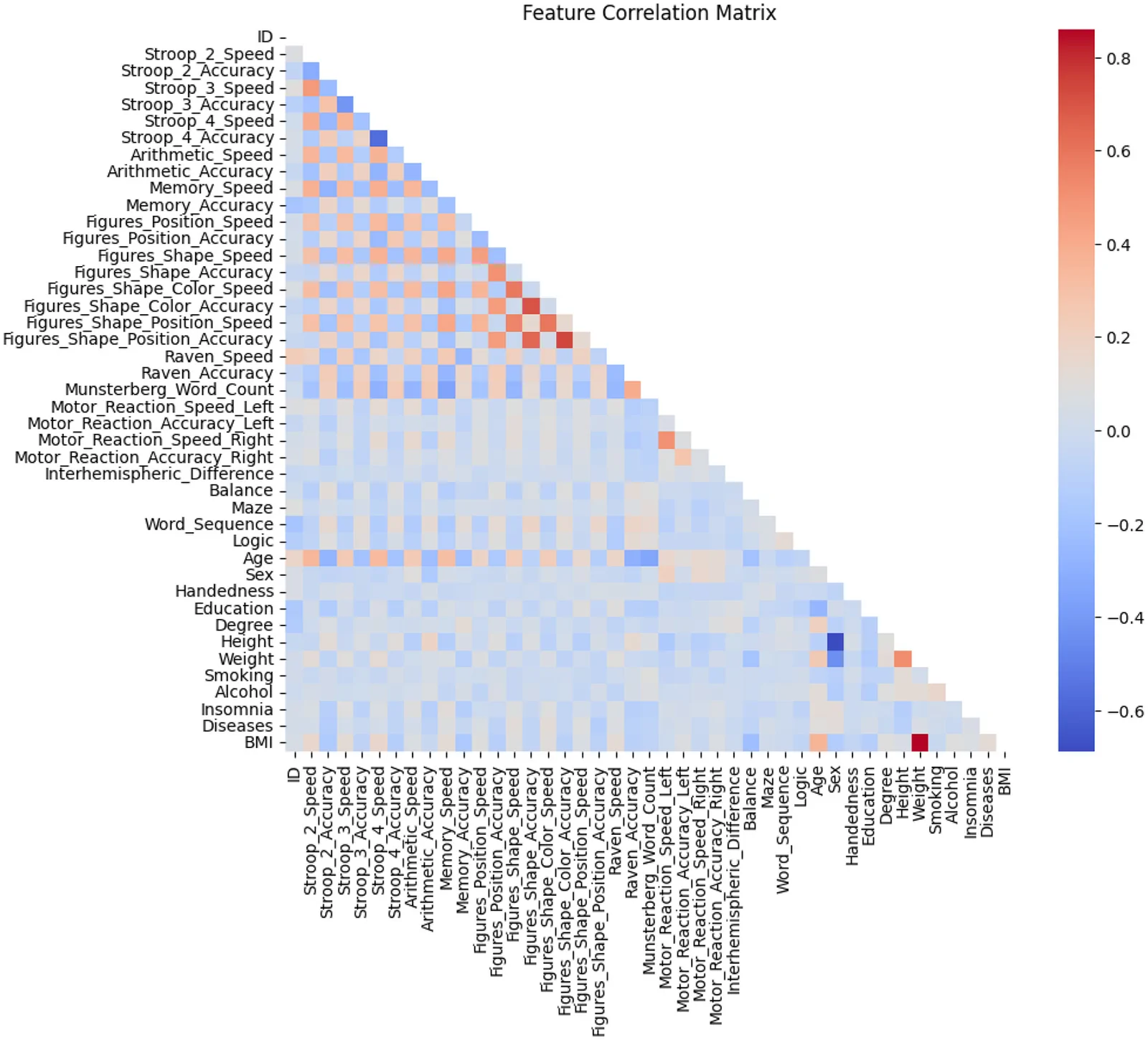
Spearman correlation matrix for psychophysiological, demographic, anthropometric, lifestyle, and health-related variables. The triangular heatmap shows the correlation structure among variables used for trajectory analysis.

Spearman correlation analysis showed that several variables measuring related cognitive domains were moderately to strongly correlated, especially accuracy measures from figure-based tasks and speed measures from related visual tasks (Figure 4). This supported the use of dimensionality reduction before elastic principal-tree construction, because the original feature space contained partially redundant information.

**Figure 5:**
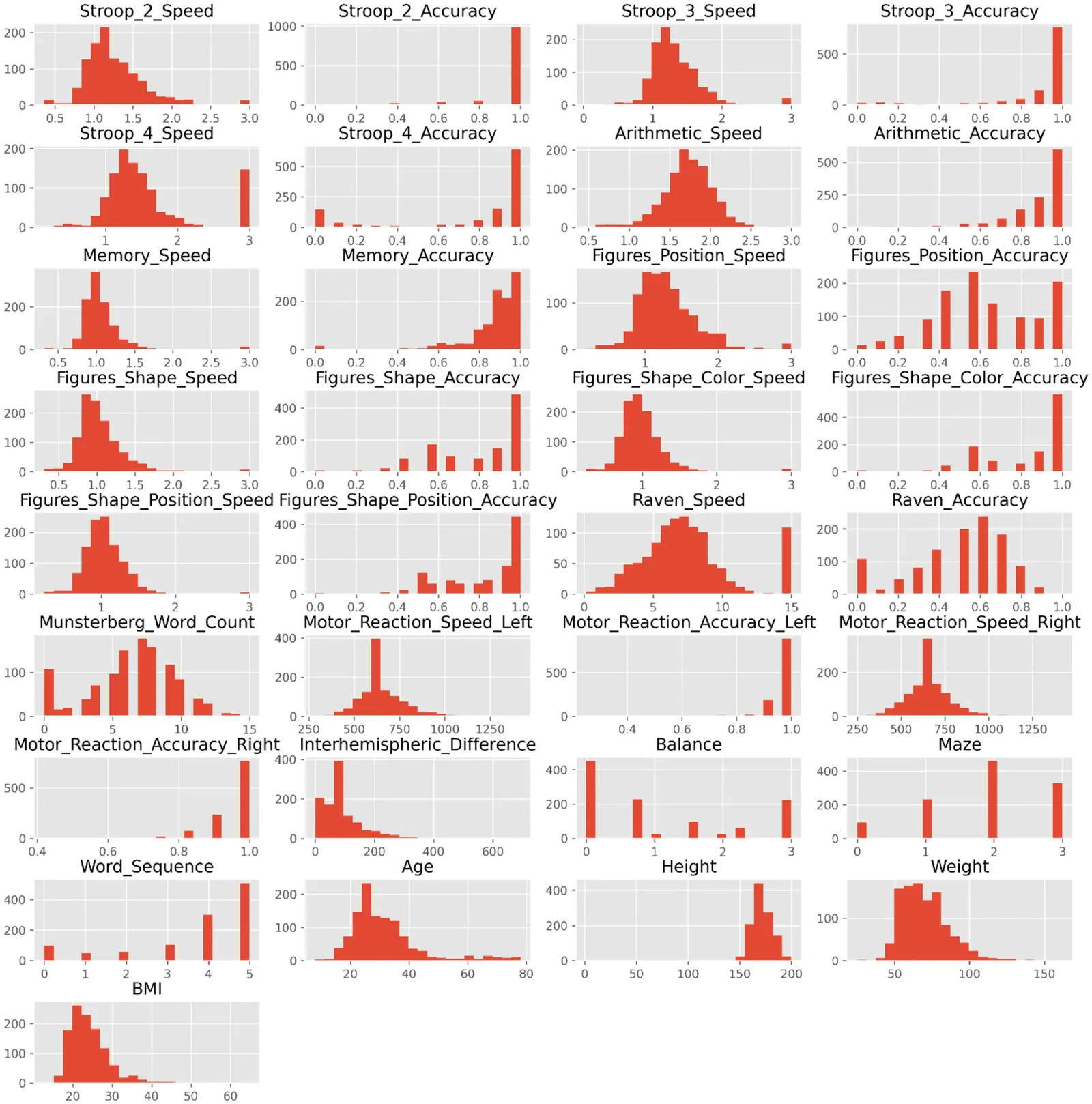
Distributions of continuous variables. Histograms show the empirical distributions of cognitive speed and accuracy measures, motor variables, age, height, weight, and BMI before trajectory interpretation.

The distributions of continuous variables showed substantial heterogeneity across tasks, with speed variables generally showing unimodal but right-skewed distributions and accuracy variables clustering close to the upper boundary for several tests (Figure 5). Age, weight, and BMI also showed right-skewed distributions. These patterns indicate that many participants performed near ceiling in accuracy-based tasks, whereas speed variables provided more continuous variation for trajectory inference.

**Figure 6:**
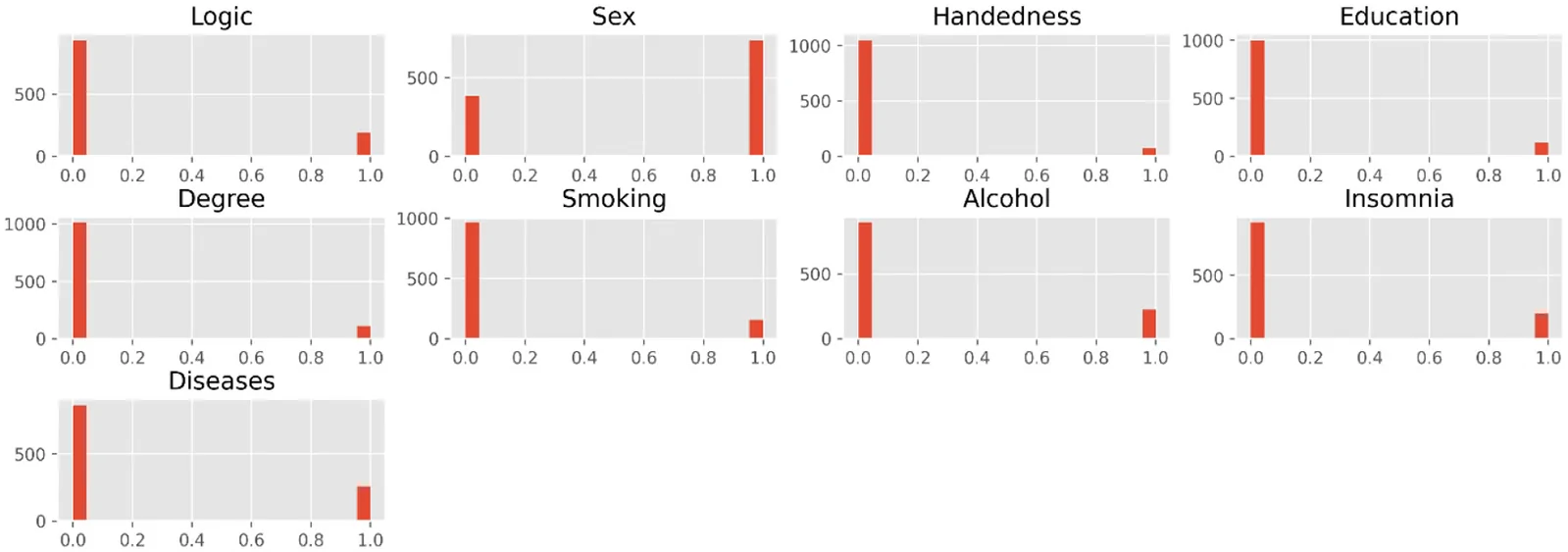
Distributions of binary variables, including logic, sex, handedness, education, degree status, smoking, alcohol use, insomnia, and disease status.

Binary variables showed uneven class proportions, as expected for demographic, lifestyle, and health-related covariates (Figure 6). Logic, handedness, education, degree status, smoking, alcohol use, insomnia, and disease status were therefore interpreted as coded binary indicators. Their 0/1 directions and interpretation limitations are summarized in Supplementary Table S3.

The numerical intrinsic-dimension estimates were 5.83 (DANCo) and 3.82 (local PCA). The first six PCs explained 39.24% of standardized variance, and their dominant loadings represented age/memory speed, anthropometry/sex, visuospatial accuracy, motor accuracy and Stroop speed, memory accuracy, and motor speed (Supplementary Table S1).

### 3.2 Sensitivity-analysis results

**Figure 7:**
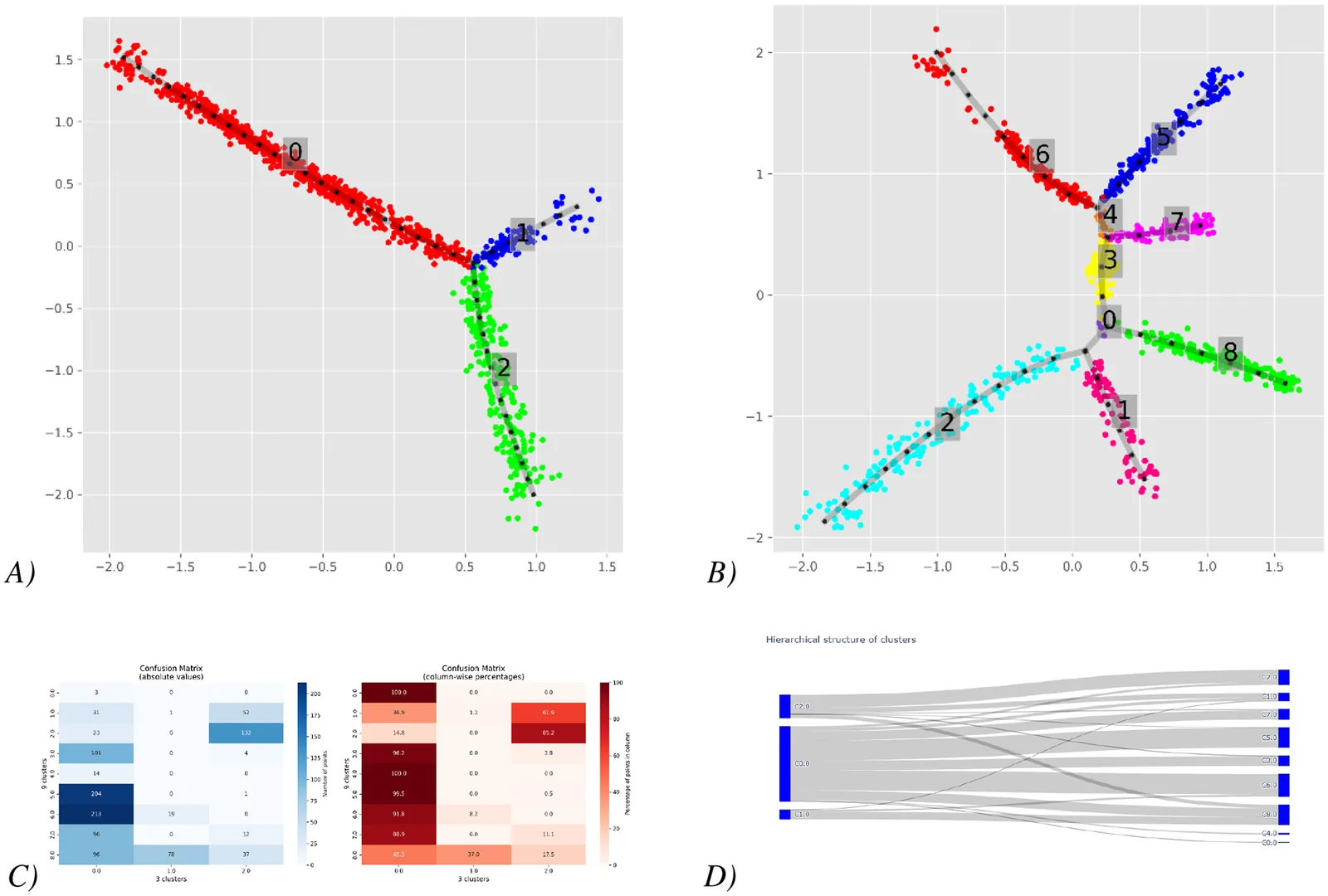
Sensitivity analysis after removing the binary variables. **(A)** Principal tree for the complete dataset: three clusters. **(B)** Principal tree after removal of binary variables: nine clusters. **(C)** Contingency matrices in absolute counts and column percentages. **(D)** Distribution of the original three clusters among the nine new clusters. Agreement with the original partition was ARI = 0.1203.

When the nine binary variables were removed, the original three-cluster solution was replaced by nine clusters (Figure 7). The agreement with the original partition was low (ARI = 0.1203), indicating that the new clusters did not coincide with the original clusters. Original Cluster 0 was distributed across the nine-cluster solution as follows: Cluster 0 (0%), Cluster 1 (4%), Cluster 2 (3%), Cluster 3 (13%), Cluster 4 (2%), Cluster 5 (26%), Cluster 6 (27%), Cluster 7 (12%), and Cluster 8 (12%). Original Cluster 1 was distributed mainly into Cluster 8 (80%) and Cluster 6 (19%), with 1% in Cluster 1. Original Cluster 2 was distributed mainly into Cluster 2 (55%) and Cluster 1 (22%), with 16% in Cluster 8, 5% in Cluster 7, 2% in Cluster 3, and less than 1% in Cluster 5. Thus, original Cluster 0 was divided mainly into Clusters 3, 5, 6, 7, and 8; original Cluster 1 moved almost entirely to Cluster 8; and original Cluster 2 moved mainly to Clusters 1 and 2. The remaining small fractions formed very small Clusters 0 and 4.

**Figure 8:**
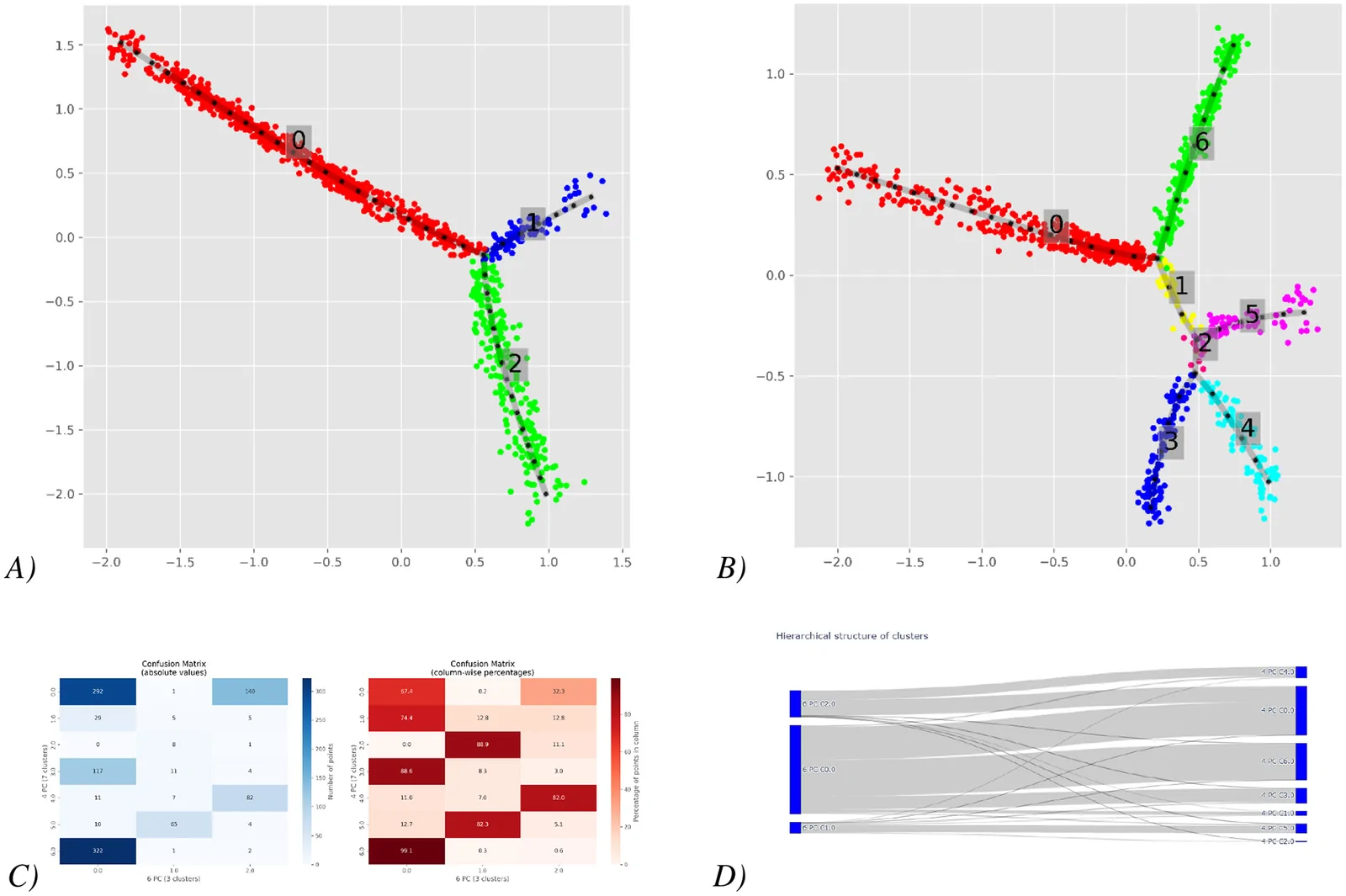
Sensitivity analysis for the number of retained PCs. **(A)** Reference principal tree using six PCs: three clusters. **(B)** Principal tree using four PCs: seven clusters. **(C)** Contingency matrices in absolute counts and column percentages. **(D)** Distribution of the original six-PC clusters among the seven four-PC clusters. Six PCs explained 39.2% of variance, four PCs explained 31.3%, and agreement was ARI = 0.1736.

The four-PC analysis also produced a more fragmented solution: the original three clusters obtained with six PCs were replaced by seven clusters, and agreement was low (ARI=0.1736; Figure 8). Original Cluster 0 was distributed as follows: Cluster 0 (37%), Cluster 1 (4%), Cluster 3 (15%), Cluster 4 (1%), Cluster 5 (1%), and Cluster 6 (41%). Original Cluster 1 was distributed across Cluster 0 (1%), Cluster 1 (5%), Cluster 2 (8%), Cluster 3 (11%), Cluster 4 (7%), Cluster 5 (66%), and Cluster 6 (1%). Original Cluster 2 was distributed across Cluster 0 (59%), Cluster 1 (2%), Cluster 2 (less than 1%), Cluster 3 (2%), Cluster 4 (34%), Cluster 5 (2%), and Cluster 6 (1%). Therefore, original Cluster 0 was divided mainly into Clusters 0, 1, 3, and 6; original Cluster 1 moved predominantly to Cluster 5; and original Cluster 2 was divided mainly into Clusters 0 and 4. Small fractions of the original clusters were mixed among these clusters and also formed the small Cluster 2.

For both sensitivity analyses, the original full-feature six-PC three-cluster structure was retained as the most interpretable solution among those examined, because removing the binary variables or reducing the number of PCs generated additional very small clusters that could not be characterized reliably (Table 2).

**Table 2:** Summary of the two additional sensitivity analyses.

Analysis	Explained variance	Number of clusters	ARI vs. reference
Full dataset, 6 PCs	39.2%	3	1.0000
Without binary variables	—	9	0.1203
Full dataset, 4 PCs	31.3%	7	0.1736

### 3.3 Elastic principal tree and branch-based clusters

**Figure 9:**
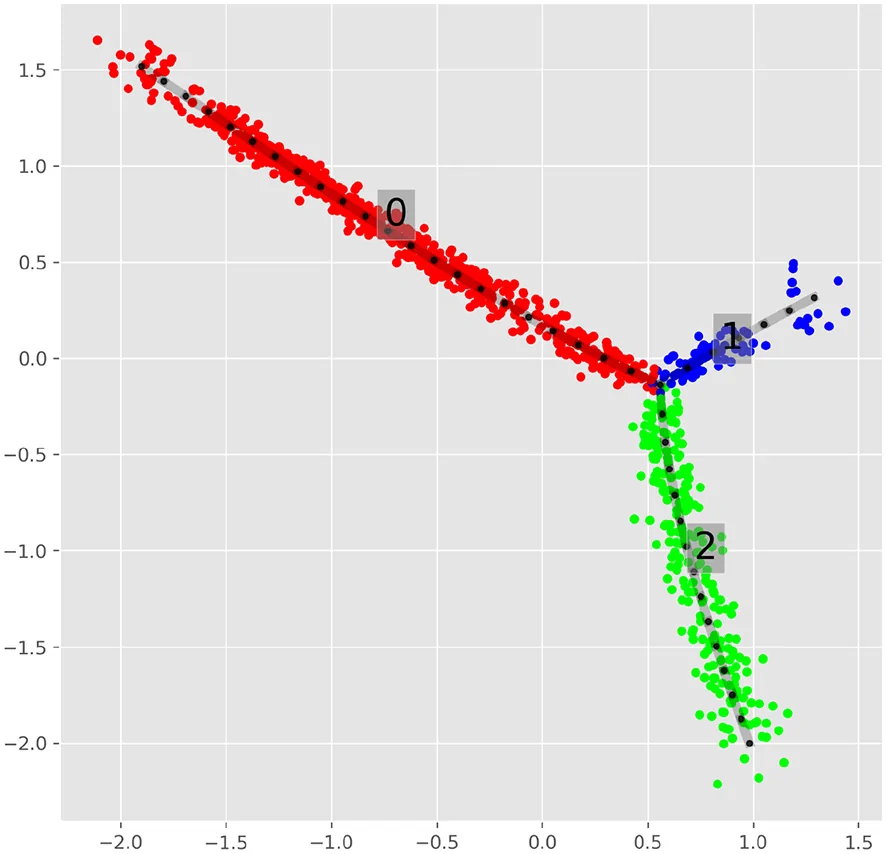
Projection of participants onto the elastic principal tree. Colors indicate branch-based clusters derived from non-branching tree segments: Cluster 0, Cluster 1, and Cluster 2. Colored points are participants, black circles are graph nodes, and gray lines are graph edges. Node symbols are displayed at a fixed size; local sample density is represented by the concentration of participant points. Root orientation is shown separately in Figure 2.

The elastic principal tree summarized the multidimensional cognitive-testing space as a branching structure and separated participants into three non-branching branch-based clusters (Figure 9). Cluster 0 contained the largest part of the sample (*n* = 781, 69.9%) and occupied the long leftward branch. Cluster 1 was the smallest group (*n* = 98, 8.8%) and occupied the short rightward branch. Cluster 2 contained 238 participants (21.3%) and occupied the downward branch. This topology indicates that the dataset was not organized as isolated groups but as a connected cognitive-performance structure with shared and diverging regions.

### 3.4 Root-node selection

Median age was compared across the three branches of the principal tree (Figure 2A), and the node-wise proportions of participants younger than 25 years were examined (Figure 2B). The right branch had the lowest median age, and Panel B showed that younger participants were concentrated along this same branch and its terminal region. In addition, the candidate root region on the right branch corresponded to participants without coded smoking, alcohol-use, insomnia, and disease indicators. These age and binary lifestyle/health characteristics jointly identified the right branch as the youngest and most favorable starting branch. Node 40 is the terminal node at its outer end and was selected as the root because this terminal position provides a suitable starting point for constructing trajectories toward the other branches. This post-fitting choice oriented the existing graph and did not determine the tree topology or cluster membership.

### 3.5 Trajectory structure

**Figure 10:**
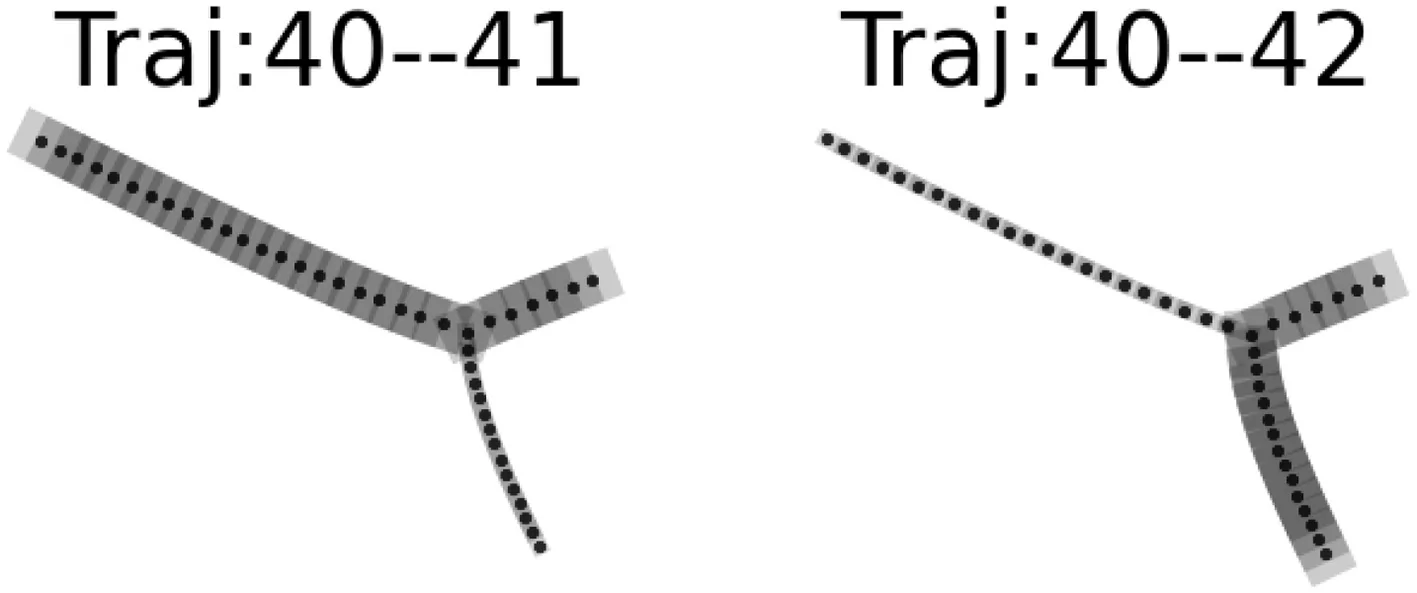
Extracted pseudo-time trajectories. Two informative trajectories were identified after root-node selection: trajectory 40–41 and trajectory 40–42. Gray shading indicates the corresponding trajectory paths on the principal tree. These are model-derived paths through cross-sectional observations, not observed within-participant temporal trajectories.

After removal of a non-informative single-branch trajectory, two main trajectories were retained for interpretation: trajectory 40–41 and trajectory 40–42 (Figure 10). Trajectory 40–41 extended from the young/right root region through the central bifurcation toward the long left branch, whereas trajectory 40–42 extended from the root through the same central region toward the downward branch. This structure suggests that participants occupy a shared relatively young region before the graph separates toward different cognitive-performance branches; it does not demonstrate within-person divergence over time.

### 3.6 Variable changes along pseudo-time

Regression of individual variables against pseudo-time was used to identify features whose values changed along each trajectory. As an example, Stroop_2_Speed increased along trajectory 40–42, with *R*^2^ = 0.38 (Figure 3). Because larger speed values correspond to longer response times, this pattern indicates slowing along this trajectory. The predicted regression curve and sliding average were consistent, supporting the interpretation of pseudo-time as a descriptive cross-sectional ordering of observed cognitive-performance variation within the branch, not as measured within-person change.

**Figure 11:**
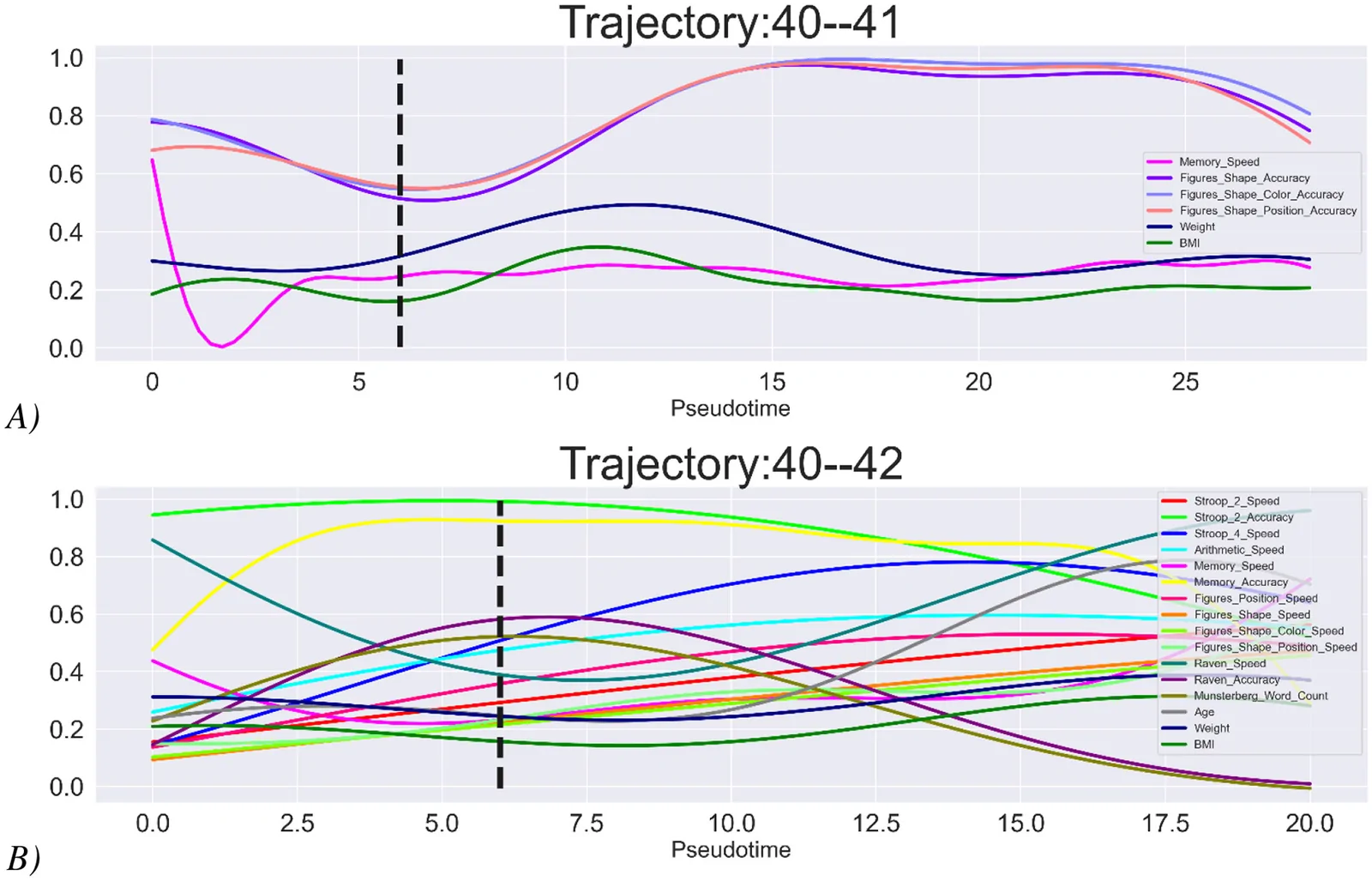
Pseudo-time dynamics of variables associated with the two main trajectories. **(A)** Trajectory 40–41. **(B)** Trajectory 40–42. Curves show normalized variable values along pseudo-time; the dashed vertical line marks the bifurcation point. The curves describe cross-sectional variation along graph distance and do not represent within-person change; formal uncertainty bands were not estimated.

The multi-variable pseudo-time profiles differed between trajectories (Figure 11). Trajectory 40–41 was characterized by a smaller set of variables with relatively stable or moderately changing values, whereas trajectory 40–42 showed broader cross-sectional variation across speed, accuracy, age, anthropometric, and health-related indicators. The dashed vertical line marks a bifurcation region, after which the trajectories become more phenotypically distinct.

### 3.7 Cluster-level phenotype interpretation

The branch-based clusters differed in their cognitive and health-related profiles. Cluster 0 showed values close to the overall dataset mean for most continuous variables, consistent with a large sample-internal reference profile. Cluster 1 showed lower-than-average speed values for several cognitive tasks, indicating faster responses, and lower BMI. Cluster 2 showed the clearest deviation from the overall mean, with higher values for several speed measures, lower values for several accuracy measures, and higher coded insomnia and disease values. Because higher speed values reflect longer response times, the Cluster 2 profile indicates generalized slowing. The profile-level results are summarized in Table 3.

**Table 3:** Summary of branch-based cognitive-performance profiles.

Profile	*n* (%)	Age, years mean ±SD	Core cognitive pattern	Health/lifestyle coded pattern
Cluster 0: high-accuracy reference profile	781 (69.9%)	29.1 ± 7.3	High accuracies in Stroop 2–4 (0.977, 0.926, 0.859), arithmetic (0.927), memory (0.895), figure-shape/color tasks (>0.87), and motor reaction (>0.96). Intermediate speeds: Stroop 4 speed 1.45; Raven speed 6.82; Munsterberg words 7.12.	Insomnia 0.156; diseases 0.186; smoking 0.160; alcohol 0.227; BMI 24.1.
Cluster 1: rapid-processing profile with domain-specific variability	98 (8.8%)	26.3 ± 5.7	Fastest task performance across Stroop, arithmetic, figure-position, shape, and shape-color tasks; Stroop 4 speed 1.16; arithmetic speed 1.49. Accuracy was high for Stroop 4 (0.930) and arithmetic (0.940), but visuospatial accuracies were lower than Cluster 0 in several figure tasks.	Insomnia 0.174; diseases 0.276; smoking 0.122; alcohol 0.184; BMI 22.4. The coded female proportion was 0.918 (0 = male, 1 = female).
Cluster 2: slower/lower-accuracy profile with older mean age and higher contextual indicators	238 (21.3%)	40.9 ± 19.2	Generalized slowing and lower accuracy. Stroop 4 speed was highest (2.16) and Stroop 4 accuracy was lowest (0.432). Arithmetic accuracy 0.789; memory accuracy 0.819; Raven speed 9.30; Raven accuracy 0.327; Munsterberg word count 4.08.	Highest coded insomnia (0.265) and diseases (0.349). Smoking and alcohol coding were lower (0.080 and 0.122), probably reflecting cohort composition or coding rather than a protective effect. BMI 24.1.

Formal comparisons confirmed that the branches differed most strongly in demanding executive and visuospatial measures. Stroop-4 speed and accuracy had eta-squared values of 0.195 and 0.194. Figure-shape, figure-shape-color, and figure-shape-position accuracy had eta-squared values of 0.240–0.253. Age differed across branches (*H* = 72.57, *p* < 0.0001, η^2^ = 0.065). Insomnia and disease indicators also differed, with small effects (Cramer's *V* = 0.114 and 0.161). Left/right motor-reaction accuracy, interhemispheric difference, maze score, and handedness did not show meaningful branch differences. Selected results are summarized in Table 4; complete omnibus and Holm-adjusted *post-hoc* results are provided in Supplementary Table S2.

**Table 4:** Selected between-branch statistical comparisons.

Variable	Statistic	*p*	Effect size	Magnitude
Stroop-4 speed	*H* = 217.96	< 0.0001	η^2^ = 0.195	Moderate/large
Stroop-4 accuracy	*H* = 216.17	< 0.0001	η^2^ = 0.194	Moderate/large
Figure-shape accuracy	*H* = 268.04	< 0.0001	η^2^ = 0.240	Large
Figure-shape-color accuracy	*H* = 279.72	< 0.0001	η^2^ = 0.251	Large
Figure-shape-position accuracy	*H* = 282.47	< 0.0001	η^2^ = 0.253	Large
Age	*H* = 72.57	< 0.0001	η^2^ = 0.065	Small/moderate
Insomnia indicator	χ^2^ = 14.53	0.0007	*V* = 0.114	Small
Disease indicator	χ^2^ = 28.90	< 0.0001	*V* = 0.161	Small

### 3.8 Cluster 0: high-accuracy reference profile

Cluster 0 showed a relatively young mean age (29.1 ± 7.3 years) and generally preserved accuracy across most cognitive tasks. Stroop accuracy remained high, especially in Stroop 2 and Stroop 3, and accuracy in arithmetic, memory, figure-shape/color tasks, and motor reaction tasks was also high. Speeds were not the fastest in the dataset, but they were substantially better than Cluster 2. The combination of high accuracy, moderate-to-good speed, lower coded insomnia, and lower coded disease burden supports interpretation of Cluster 0 as a high-accuracy reference profile within this sample.

### 3.9 Cluster 1: rapid-processing profile with domain-specific variability

Cluster 1 was the youngest group (26.3 ± 5.7 years) and showed the fastest mean performance on several speed measures, including Stroop 2, Stroop 3, Stroop 4, arithmetic, figure-position, figure-shape, and figure-shape-color tasks. This profile is consistent with a rapid-processing pattern in this sample. However, some visuospatial accuracies were lower than in Cluster 0, suggesting that this branch should not be interpreted as globally superior. Instead, it appears to represent a high-speed profile with domain-specific variability. Because this cluster is small, interpretation of its demographic composition should remain cautious despite the documented coding directions.

### 3.10 Cluster 2: slower/lower-accuracy profile with older mean age and higher contextual indicators

Cluster 2 had the highest mean age (40.9 ± 19.2 years) and the widest age dispersion, indicating substantial internal age heterogeneity rather than a uniform older group. The cognitive signature of this cluster was consistent across domains: slower Stroop responses, markedly lower Stroop 4 accuracy, slower arithmetic, lower arithmetic accuracy, slower and less accurate memory performance, lower visuospatial accuracy, slower Raven task performance, lower Raven accuracy, fewer Munsterberg words, and slightly lower motor-reaction accuracy. The most pronounced contrast was in the cognitively demanding Stroop 4 condition, where Cluster 2 had much slower performance and substantially lower accuracy than Clusters 0 and 1. This pattern describes lower performance in executive-control, processing-speed, and visual/verbal tasks within this sample.

### 3.11 Health and lifestyle covariates

Cluster 2 also showed the highest coded proportions for insomnia and diseases. These indicators provide contextual information alongside the cognitive-performance differences but do not establish a clinical or risk phenotype. Smoking and alcohol coding were lower in Cluster 2 than in the younger clusters, but the analysis does not justify interpreting these lower values as protective. The supplied data do not include dose, duration, former-use history, or medical contraindications; therefore, lifestyle variables should be treated as contextual covariates rather than causal explanations. BMI was similar in Clusters 0 and 2 and lower in Cluster 1.

### 3.12 Descriptive comparison of branch profiles

Taken together, Cluster 0 represents a high-accuracy reference profile, Cluster 1 a rapid-processing profile with task-specific variability, and Cluster 2 a slower/lower-accuracy profile with older mean age and higher coded insomnia/disease indicators. These are descriptive partitions of the present sample, not healthy-versus-atypical aging classes or clinical diagnoses. The wide age dispersion in Cluster 2 further precludes interpreting it as a uniform older or pathological subgroup.

### 3.13 Node-count complexity and assignment stability

**Figure 12:**
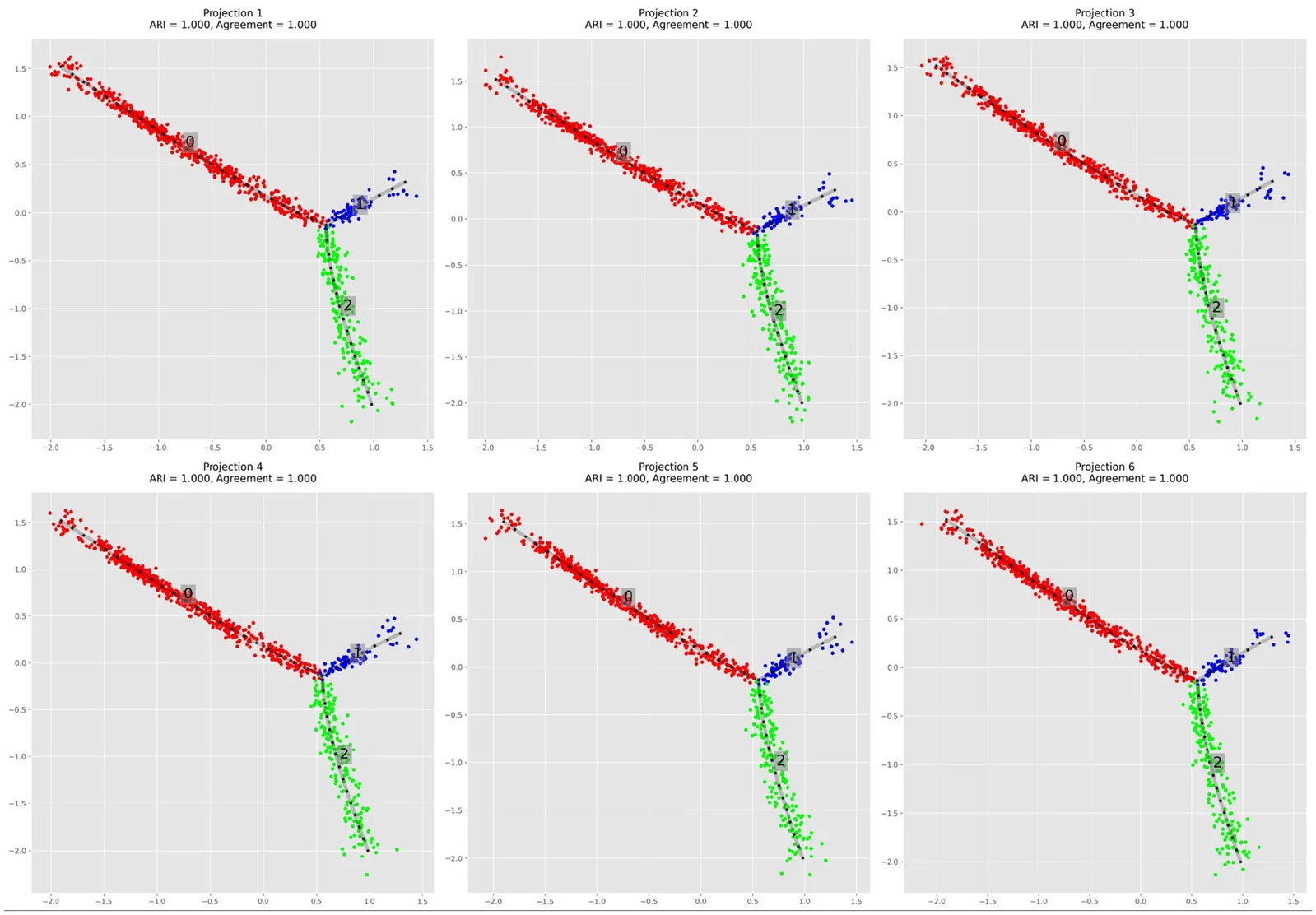
Examples of fixed-tree projection stability. Six representative random 80% subsamples were projected onto the reference tree. Colors indicate the three original branch assignments; each projection yielded ARI = 1.000 and raw agreement = 1.000. This evaluates assignment reproducibility conditional on the fixed reference graph and not stability of independently refitted topology.

The URN2 curve changed from gradual to steeper complexity growth at approximately 40 nodes (Figure 11), supporting 40 as an elbow resolution rather than an arbitrary maximum-detail choice. Across 100 random 80% subsamples, projection onto the fixed reference tree produced mean ARI 1.000 ± 0.000 (95% interval 1.000–1.000), mean NMI 1.000 ± 0.000, and mean raw agreement 1.000 ± 0.000. The first six projection examples are shown in Figure 12; each reproduced the reference branch assignment. These findings demonstrate deterministic assignment stability conditional on the fitted tree, not identical topology under independent graph refitting or external validation.

### 3.14 Heatmap visualization of cluster-specific Cognitive-performance profiles

**Figure 13:**
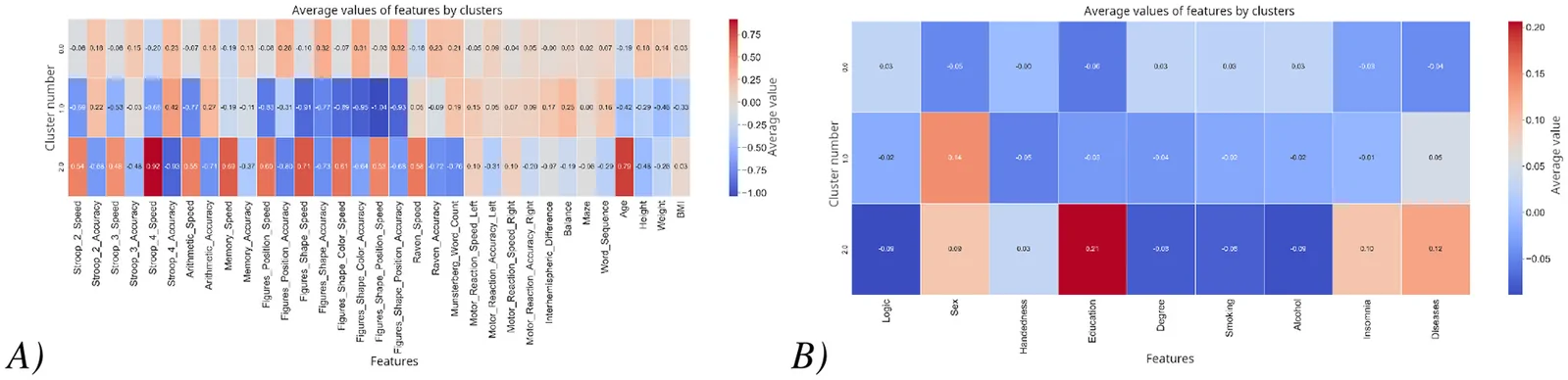
Heatmap of mean feature values by cluster. **(A)** Continuous variables. **(B)** Binary variables. Colors represent relative mean values across clusters, highlighting variables that differentiate the branch-based cognitive-performance profiles. The display is descriptive and does not imply clinical classes.

Heatmap visualization of standardized cluster means confirmed the separation of the three profiles (Figure 13). Cluster 0 was near the center of the overall distribution across many variables, whereas Clusters 1 and 2 showed more distinctive patterns. Cluster 1 was characterized by relatively fast cognitive performance and younger age. Cluster 2 showed higher age, slower Stroop and Raven performance, reduced accuracy in demanding tasks, and higher coded values for insomnia and disease status.

Taken together, the results indicate that adult cognitive-performance variation in this dataset forms a connected branching structure rather than a single linear ordering. The root region was enriched for younger participants, and two main cross-sectional graph paths extended from this region. Cluster 0 reflected stable high-accuracy performance, Cluster 1 reflected rapid performance with task-specific variability, and Cluster 2 was characterized by older mean age, generalized slowing, poorer accuracy, lower Raven and Munsterberg performance, and higher coded insomnia/disease indicators.

## 4 Discussion

The present analysis shows that elastic principal-tree modeling can organize cognitive-test data into descriptively interpretable adult cognitive-performance profiles. The tree was oriented using the youngest branch of the data, producing a pseudo-time framework in which deviations from the root represent alternative graph paths through the observed cross-sectional structure. Specifically, the right branch had the lowest median age, the greatest concentration of participants younger than 25 years, and a candidate root region without coded smoking, alcohol-use, insomnia, and disease indicators; these age-associated lifestyle and health factors are related to cognitive functions and psychophysiological test performance. Node 40 was selected because it is the terminal node of this branch and therefore provides a logical starting point for paths toward the other branches. This is conceptually aligned with the broader trajectory approach used in ClinTrajan and in recent clinical applications of elastic principal graphs, where cross-sectional data are used to infer connected phenotypes and model-derived paths rather than disconnected clusters (Golovenkin et al., 2020; Bell et al., 2025).

Methodologically, the analysis follows the logic of dynamic phenotyping described by Golovenkin et al. (Golovenkin et al., 2020). The advantage of this strategy is that it preserves both grouping and ordering information: participants are partitioned by non-branching tree segments, but the segments remain connected through branching points. For adult cognitive-performance phenotyping, such branching points are useful because they summarize regions where participants with similar profiles occupy different connected branches. This preserves geometric information that is not represented by a purely supervised age-prediction model or a static cluster solution, without implying observed biological transitions.

The main substantive finding is the separation of a large high-accuracy reference profile and a smaller rapid-processing profile from a slower, lower-accuracy profile with older mean age. This pattern is consistent with modern views of cognitive aging as heterogeneous and domain-specific. Healthy aging does not require maximal speed in every task; rather, it can be characterized by maintained accuracy and sufficient cognitive control despite some age-related slowing. Cluster 0 therefore provides a sample-internal high-accuracy reference, while Cluster 1 captures a younger, faster profile with domain-specific variability.

Cluster 2 is the most descriptively distinct branch. Its pattern of generalized slowing and lower accuracy was most visible in executive-conflict and visuospatial tasks, especially the Stroop 4 condition. This aligns with recent evidence that conflict-control paradigms are sensitive to the transition from healthy aging to impairment (Cipriani et al., 2025; Zhu et al., 2025). The increased disease and insomnia coding in this cluster also fits recent literature showing that sleep disturbance, chronic illness, and lifestyle factors can contribute to cognitive vulnerability (Li R. et al., 2025; Zhang et al., 2025; Topiwala et al., 2026). Its age standard deviation of 19.2 years also indicates substantial internal heterogeneity, so it should not be treated as a uniform older or clinical subgroup. However, this analysis is not causal: it cannot establish whether disease, insomnia, or other factors produced cognitive slowing, whether cognitive decline changed lifestyle reporting, or whether the associations reflect age and cohort composition.

The trajectory approach adds value over a simple age regression. A cognitive-age model estimates how old a participant appears from psychophysiological tests, whereas the principal-tree model shows where the participant lies in a multidimensional cognitive space and which connected performance branch they resemble. In future work, the two approaches should be combined: supervised cognitive-age prediction can estimate cognitive-age gap, while principal-tree pseudo-time can describe which performance and contextual dimensions vary along cross-sectional graph paths.

The method also adds information beyond conventional discrete clustering. K-means or hierarchical clustering can partition participants but does not directly encode a shared central region, a bifurcation, or geodesic ordering. Hidden Markov and growth-mixture models are valuable for repeated measures but generally require longitudinal observations and transition assumptions. The present principal tree is therefore best viewed as an exploratory connected map for cross-sectional data. A new participant can be projected onto the reference graph and compared with nearby profiles, but this operation is not a clinical diagnosis.

The two sensitivity analyses produced additional small clusters: nine clusters after removal of the binary variables and seven clusters after reduction to four PCs. The low ARI values show that these partitions do not coincide with the original three-cluster partition. For the binary-variable analysis, this difference is substantively expected because the complete binary-variable block was removed. All binary features in the dataset are directly or indirectly related to cognitive functions or to the interpretation of psychophysiological test results. Their joint exclusion therefore changes the substantive interpretation of the analysis, especially because the removed block includes age-associated lifestyle and health factors such as smoking, alcohol use, insomnia, and disease status. The low ARI consequently indicates that the complete binary-variable block contributes materially to the full-feature tree structure. The original full-feature six-PC solution was retained because it corresponds to the study objective and because the alternative solutions also included very small clusters that could not be described reliably.

### 4.1 Limitations

The design is cross-sectional, so graph distance is not time and no within-person change was observed. Because the graph was oriented using age after fitting, the root cannot independently validate an aging direction. Cluster 2 is internally heterogeneous, and the smooth pseudo-time curves are descriptive; formal uncertainty bands were not estimated. Recruitment dates, ethnicity, medications, and formal neurological or psychiatric screening were not retained in the analytical export. Health variables are coarse self-reported indicators. Their coding directions are documented in Supplementary Table S3, but disease type, severity, medication, and duration remain unavailable. The six PCs explained less than half of total variance, and standard PCA is suboptimal for mixed data. In the targeted sensitivity analyses, exclusion of the binary-variable block yielded nine clusters (ARI = 0.1203) and reduction to four PCs yielded seven clusters (ARI = 0.1736). All binary features are directly or indirectly related to cognitive functions or to the interpretation of psychophysiological test results; therefore, removing the entire binary-variable block changes the substantive interpretation of the analysis, especially because the block includes age-associated lifestyle and health factors such as smoking, alcohol use, insomnia, and disease status. The full-feature solution was therefore retained as the primary model; the low ARI documents the contribution of the binary variables to the tree structure, while the additional very small clusters limit reliable interpretation of the alternative partition.

Despite these limitations, the results support the methodological premise that adult cognitive-performance variation in this dataset can be summarized by a connected branching structure rather than a single linear ordering. Cluster 2 is a descriptively distinct and internally heterogeneous group that warrants longitudinal and external study, not a validated screening or intervention target. The method is especially relevant for scalable digital cognitive testing because it can summarize many heterogeneous features into interpretable phenotypes while preserving branch structure.

## 5 Conclusion

Elastic principal-tree analysis organized the 42-variable psychophysiological dataset into three connected adult cognitive-performance profiles: a high-accuracy reference branch, a rapid-processing branch with domain-specific variability, and a slower/lower-accuracy branch enriched for older age and positive insomnia/disease indicators. Between-branch differences were statistically strongest for Stroop-4 and figure-based accuracy measures. A 40-node resolution was supported by the onset of steeper graph-complexity growth, and projection of 100 random 80% subsamples onto the fixed reference tree reproduced branch assignments perfectly conditional on the selected reference graph.

These findings support a reproducible descriptive phenotyping framework rather than cognitive-aging stages or clinical diagnoses. Because the data are cross-sectional, graph orientation and graph distance represent model-derived ordering and should not be interpreted as observed within-person progression. The additional analyses showed that removal of the binary-variable block and reduction to four PCs generated more fragmented solutions with nine and seven clusters, respectively. All binary features are directly or indirectly related to cognitive functions or to the interpretation of psychophysiological test results. Removing the entire binary-variable block therefore changes the substantive interpretation of the analysis, particularly because the block includes age-associated lifestyle and health factors such as smoking, alcohol use, insomnia, and disease status. The original full-feature six-PC three-cluster solution was therefore retained as the primary model. Longitudinal follow-up, older and clinically characterized cohorts, mixed-data sensitivity analyses, and independent-cohort projection are required for biological or clinical validation.

## Data Availability

The data that support the findings of this study are available from the corresponding author upon reasonable request. Analysis code, notebooks, the repository dataset, and statistical-test scripts are available at https://github.com/AlexanderZapryalov/cognitive-tests. The binary-variable coding dictionary is also provided with the manuscript as Supplementary Table S3.
